# Detection of Circulating Tumor DNA in Patients With Uterine Leiomyomas

**DOI:** 10.1200/PO.18.00409

**Published:** 2019-10-16

**Authors:** Joanna Przybyl, Lien Spans, Deirdre A. Lum, Shirley Zhu, Sujay Vennam, Erna Forgó, Sushama Varma, Kristen Ganjoo, Trevor Hastie, Raffick Bowen, Maria Debiec-Rychter, Matt van de Rijn

**Affiliations:** ^1^ Stanford University School of Medicine, Stanford, CA; ^2^ KU Leuven and University Hospitals Leuven, Leuven, Belgium; ^3^ Stanford University, Stanford, CA

## Abstract

**PURPOSE:**

The preoperative distinction between uterine leiomyoma (LM) and leiomyosarcoma (LMS) is difficult, which may result in dissemination of an unexpected malignancy during surgery for a presumed benign lesion. An assay based on circulating tumor DNA (ctDNA) could help in the preoperative distinction between LM and LMS. This study addresses the feasibility of applying the two most frequently used approaches for detection of ctDNA: profiling of copy number alterations (CNAs) and point mutations in the plasma of patients with LM.

**PATIENTS AND METHODS:**

By shallow whole-genome sequencing, we prospectively examined whether LM-derived ctDNA could be detected in plasma specimens of 12 patients. Plasma levels of lactate dehydrogenase, a marker suggested for the distinction between LM and LMS by prior studies, were also determined. We also profiled 36 LM tumor specimens by exome sequencing to develop a panel for targeted detection of point mutations in ctDNA of patients with LM.

**RESULTS:**

We identified tumor-derived CNAs in the plasma DNA of 50% (six of 12) of patients with LM. The lactate dehydrogenase levels did not allow for an accurate distinction between patients with LM and patients with LMS. We identified only two recurrently mutated genes in LM tumors (*MED12* and *ACLY*).

**CONCLUSION:**

Our results show that LMs do shed DNA into the circulation, which provides an opportunity for the development of ctDNA-based testing to distinguish LM from LMS. Although we could not design an LM-specific panel for ctDNA profiling, we propose that the detection of CNAs or point mutations in selected tumor suppressor genes in ctDNA may favor a diagnosis of LMS, since these genes are not affected in LM.

## INTRODUCTION

Uterine leiomyomas (LMs) are common benign smooth muscle tumors that may present with symptoms similar to those associated with uterine leiomyosarcoma (LMS), a rare malignant tumor with a poor prognosis.^1^ With the exception of endometrial lesions, most uterine masses are not biopsied before surgery, and the preoperative distinction between benign and malignant uterine smooth muscle tumors relies primarily on clinical evaluation and imaging. As a result, patients may undergo surgery without a definitive distinction between the two entities.

It is estimated that one in nine women in the United States will undergo a hysterectomy for benign gynecologic indications, such as LM, in their lifetime.^2^ Power morcellation used to be performed in many such cases, until in 2014 the US Food and Drug Administration discouraged the use of power morcellation for the removal of the uterus or uterine masses, after reporting that this procedure may lead to inadvertent retroperitoneal spread of an unsuspected malignancy in one in 305 patients.^3^ However, intra-abdominal manual morcellation is still performed in patients with large masses diagnosed as LM, and although this is less aggressive than power morcellation, it still carries a risk for dissemination of an unexpected LMS. Risk factors that may favor the diagnosis of LMS over LM include postmenopausal status, tamoxifen use, history of retinoblastoma, pelvic irradiation, hereditary leiomyomatosis, and renal cell cancer syndrome,^4^ but these factors do not always correlate with a diagnosis of LMS. Also, the new emerging magnetic resonance imaging techniques present unsatisfactory positive predictive values for the distinction between LM and LMS.^4^ Therefore, there is a high need for improved methods for preoperative discrimination between benign LM and malignant tumors. In an attempt to improve the distinction between LM and LMS, Nagai et al^5^ developed a “revised preoperative sarcoma score” (rPRESS) based on patient’s age, serum lactate dehydrogenase (LDH) levels, and endometrial cytology findings. This system was developed in a group of 63 patients with LM and LMS but has not yet been validated in an independent study.

**Context**

**Key Objective**
We sought to explore the feasibility of distinguishing between uterine leiomyoma and leiomyosarcoma based on the analysis of tumor-specific aberrations in circulating tumor DNA (ctDNA). For this purpose, in the current study we aimed to determine whether uterine leiomyomas shed DNA into the circulation.
**Knowledge Generated**
We demonstrate that it is feasible to detect ctDNA derived from leiomyomas. In the limited number of cases analyzed to date, we show that these benign tumors do not carry alterations in tumor suppressor genes that can be detected in the ctDNA of most patients with leiomyosarcoma.
**Relevance**
Our findings are a first and necessary step toward developing a blood test that, together with clinical and imaging information, ultimately could help clinicians to better evaluate the risk of leiomyosarcoma in a patient presenting with a uterine mass.

We recently demonstrated that tumor-associated genetic aberrations can be detected in the circulating tumor DNA (ctDNA) of patients with LMS.^6^ In that study, we used two technologies to detect different classes of genomic aberrations in plasma DNA: cancer personalized profiling by deep sequencing (CAPP-Seq) for the analysis of point mutations,^7,8^ and a genome-wide interrogation of copy number alterations (CNAs) by shallow whole-genome sequencing.^9,10^ Using these two approaches, we were able to detect ctDNA in six of seven patients with LMS with > 98% specificity for mutant allele fractions as low as 0.01%. We hypothesized that if LM nodules also shed DNA into the circulatory system, an approach based on ctDNA profiling could be useful for a distinction between LM and LMS using blood samples.

## PATIENTS AND METHODS

### Patients With LM

The clinical features of 12 patients with LM who provided blood specimens for this study are listed in Table 1. The study was approved by the Stanford University Institutional Review Board (IRB-31067).

**TABLE 1.**
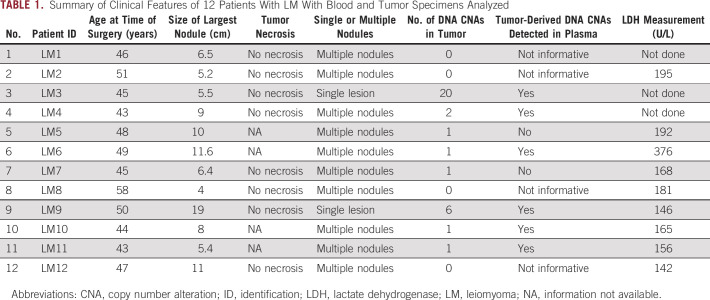
Summary of Clinical Features of 12 Patients With LM With Blood and Tumor Specimens Analyzed

### Profiling Plasma Cell-Free DNA

Cell-free DNA was extracted from plasma using QIAamp Circulating Nucleic Acid kit (Qiagen). Sequencing libraries were prepared with the TruSeq ChIP preparation kit (Illumina, Foster City, CA) and sequenced using HiSeq 2500 instrument (Illumina) (Data Supplement).

### Plasma LDH Measurement

Plasma LDH levels were measured on a Roche Cobas 8000 (Roche, Indianapolis, IN) automated platform (Data Supplement).

### Profiling Tumor Specimens

Genomic DNA extracted from 12 archival LM tumor specimens and germline DNA extracted from patient-matched blood leukocytes were used for genome-wide copy number profiling with the OncoScan FFPE Assay (Thermo Fisher Scientific/Affymetrix, Santa Clara, CA).

Thirty-six archival LM tumor specimens and normal patient-matched counterpart tissues or peripheral blood leukocytes were used for whole-exome sequencing using the SeqCap EZ Human Exome v3.0 Library (Roche) on HiSeq 4000 instrument (Illumina) (Data Supplement).

## RESULTS

### Tumor-Derived Copy Number Aberrations Can Be Detected in Plasma DNA of Patients With LM

**FIG A1.**
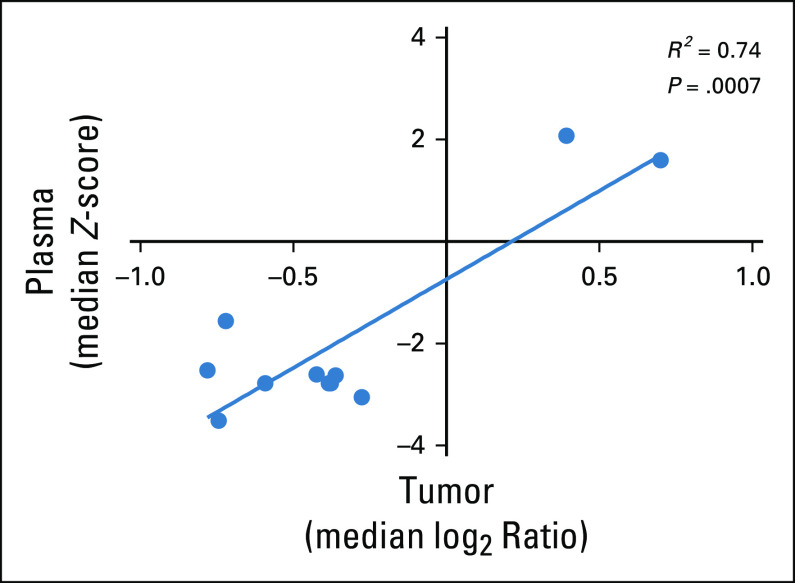
Correlation between log_2_ ratio in tumor DNA and Z-score in cell-free DNA for 11 tumor-derived copy number alterations detected in plasma of leiomyoma patients. Correlation was determined by linear regression (*R*^*2*^).

To explore the possibility of developing a blood-based test for the distinction between LM and LMS, we first determined whether LM can shed DNA in the blood. We characterized LM tumors from 12 patients by profiling CNAs using single-nucleotide polymorphism arrays. We also profiled CNAs in patient-matched germline DNA extracted from peripheral blood leukocytes to exclude structural polymorphisms from tumor profiles. With this approach, we found 33 somatic CNAs in eight of 12 LM tumor specimens, with a median of one CNA per tumor (range, 1-20 in the eight LM tumor specimens; Table 2). Next, we profiled CNAs in the plasma DNA of these 12 patients with LM by shallow whole-genome sequencing. The median genome-wide coverage of the uniquely mapped deduplicated reads was 0.1× (range, 0.08×-0.12×; Data Supplement). Specifically, we sought to investigate whether CNAs found in LM tumor specimens can be also found in plasma cell-free DNA of the same patient. We calculated genome-wide segmented *Z*-scores in plasma cell-free DNA, and we intersected each genomic region of CNAs identified in the eight LM tumor specimens with the segments identified in plasma DNA of the matching patients. Of the 33 CNAs detected in the eight tumor specimens, 30 CNAs had the same type of copy number alteration in tumor and plasma DNA (ie, matching DNA copy number gain or loss; Data Supplement). When we limited these aberrations to those with the *Z*-score < −1.5 and > 1.5 in cell-free DNA, we found 11 tumor-derived CNAs in the plasma DNA of six patients with LM (correlation between log_2_ ratio in the tumor and *Z*-score in the plasma determined by linear regression was *R^2^* = 0.74; *P* value = .0007; Table 3; Appendix Fig A1). Selected large DNA CNAs identified in LM tumor specimens were not detected in plasma specimens, which is most likely the result of the limit of detection of our method and/or the effect of stochastic sampling of blood specimens. The percent of the whole genome affected by tumor-derived aberrations in plasma of these six patients was in the range of 0.001%-1.6% (Table 4). These levels were lower compared with the percent of genome altered that we had previously detected in patients with LMS, with a range of 0.05%-30.12%.^6^

**TABLE 2.**
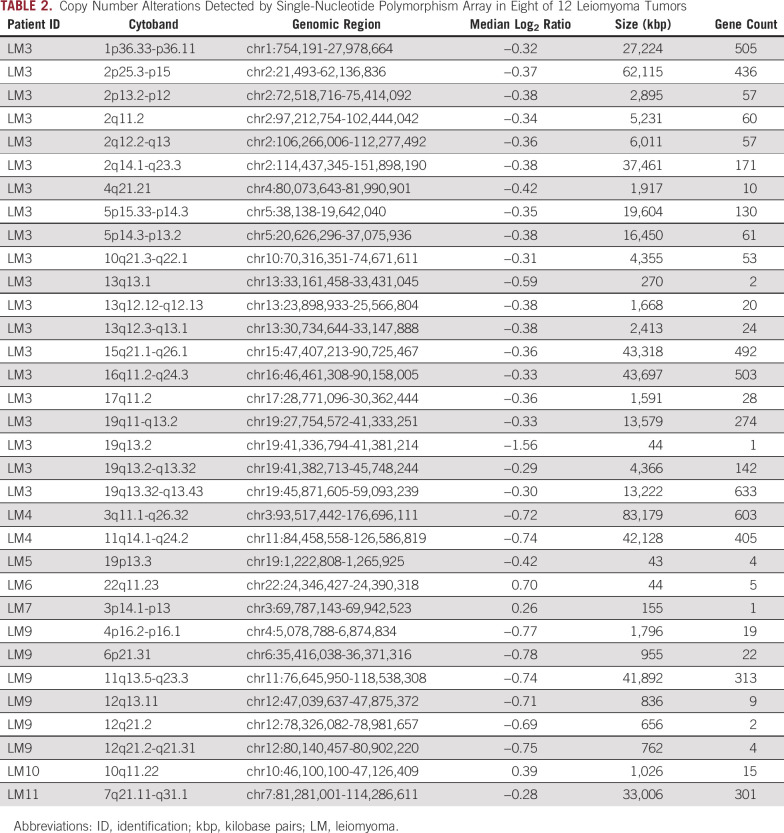
Copy Number Alterations Detected by Single-Nucleotide Polymorphism Array in Eight of 12 Leiomyoma Tumors

**TABLE 3.**
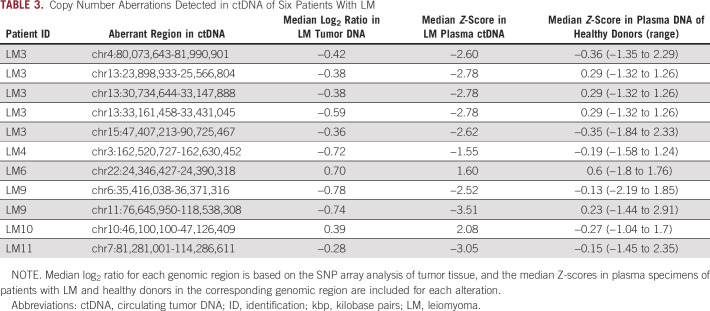
Copy Number Aberrations Detected in ctDNA of Six Patients With LM

**TABLE 4.**
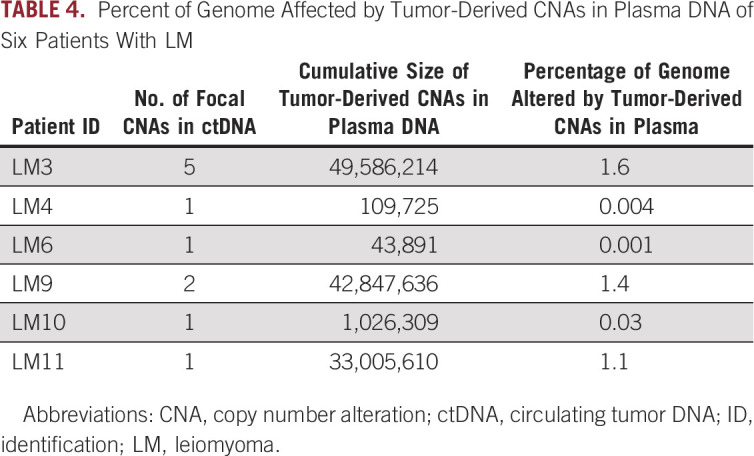
Percent of Genome Affected by Tumor-Derived CNAs in Plasma DNA of Six Patients With LM

**FIG 1.**
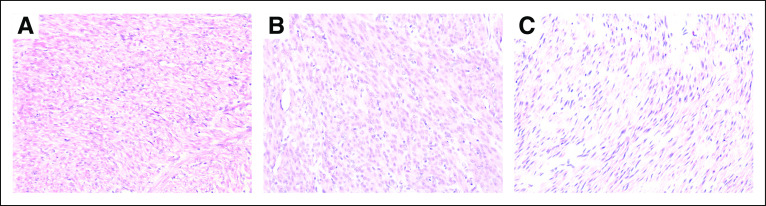
Histologic appearance of representative leiomyoma (LM) cases with (A) no detectable DNA copy number alterations, (B) 20 DNA copy number alterations, and (C) six DNA copy number alterations. Total original magnification 200×.

A review of the LM histology did not identify any differences in morphology, cellularity, or mitotic activity between the tumors of patients with detectable and undetectable CNAs in tumor and plasma specimens (Fig 1). All 11 CNAs identified in the ctDNA were unique to each patient with LM. Cell-free DNA profiles from plasma specimens of 25 healthy male donors were used to determine the specificity of detection of these 11 LM-derived CNAs. With the *Z*-score cutoff of −1.5/+1.5, the overall specificity in healthy male donors was 76% (19 of 25 healthy donors were true negative). Overall, in healthy donors, the median *Z*-scores in the regions of aberrant ctDNA identified in patients with LM were within the range of −0.36 to 0.6 (Table 3).

Selected CNAs detected in the matching plasma and tumor specimens from patients with LM were reported in DGV (accessed on June 15, 2019; Table A4). Nevertheless, because these CNAs were not present in the germline DNA of the patients with LM enrolled in the current study, these alterations represent the true tumor-derived CNAs in these specific plasma specimens.

### Overlapping CNAs Between LM and LMS

LMS is typically characterized by high genomic instability that results in extensive and heterogeneous CNAs. We have previously demonstrated that it is possible to detect tumor-derived CNAs in cell-free DNA among patients with LMS using the same technology as applied to the patients with LM in the current study.^6^ We sought to verify whether the aberrations found in ctDNA of patients with LM in the present report are specific to these benign tumors or whether the same aberrations could be found also in LMS tumor specimens. For this purpose, we reanalyzed the previously generated genomic profiles of 22 tumors from seven patients with LMS using exactly the same criteria as were used for the LM tumor specimens. Our results show that 10 of 11 genomic aberrations found in the ctDNA of patients with LM were also present in the tumor DNA of patients with LMS analyzed in our previous study,^6^ with a median of four aberrations per LMS tumor sample (range, 1-8). These results demonstrate that the majority of CNAs found in LM tumors are not specific for this entity; therefore, profiling these CNAs in plasma DNA may not be useful to distinguish between LM and LMS. On the other hand, CNAs found in LM specimens did not affect any of the frequently deleted tumor suppressor genes in LMS (eg, *TP53*, *RB1*, *ATRX*, *PTEN*, *ATM*, or *CDKN2A*). Therefore, the detection of CNAs in these tumor suppressor genes in ctDNA may favor a diagnosis of LMS.

### Patient Age and Levels of LDH Do Not Allow for Distinction Between LM and LMS

**FIG 2.**
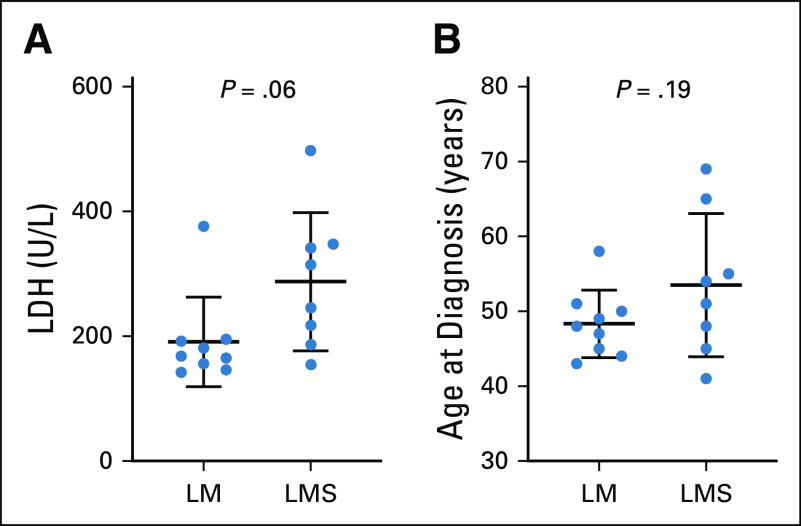
(A) Lactate dehydrogenase (LDH) levels, and (B) age of patients with leiomyoma (LM) and leiomyosarcoma (LMS). The scatter plot represents median LDH levels and age in nine patients with LM and eight patients with LMS, and the lower and upper bars represent 95% CIs. *P* values were calculated using unpaired *t* test with Welch’s correction.

Previous studies have proposed that LDH level measurements and patient age may be helpful in the distinction between LM and LMS.^5,11^ We evaluated the LDH levels in plasma specimens of nine of 12 patients with LM profiled for the presence of ctDNA and eight untreated patients with uterine LMS. The mean LDH levels were 191 and 288 U/L in the patients with LM and LMS, respectively (*t* test two-tailed *P* value = .06; Fig 2A). In the rPRESS algorithm, the cutoff of serum LDH levels indicative of LMS was set at ≥ 279 U/L.^5^ In our group of patients, the sensitivity and specificity values of this test were found to be only 50% and 89%, respectively. The positive predictive value and negative predictive value were 80% (95% CI, 35.73% to 96.64%) and 66.67% (95% CI, 49.07% to 80.59%), respectively. These results show worse performance of the LDH measurement for the distinction between LM and LMS compared with the rPRESS study, where the sensitivity, specificity, positive predictive value, and negative predictive value were 47%, 100%, 100%, and 85.7%, respectively.^5^

The rPRESS study also took patient age into account as a potential risk factor for LMS. In our small series, we did not observe a significant age difference between the 11 patients with LM and eight patients with LMS. The mean age of the patients with LM and LMS was 48 and 54 years, respectively (*t* test two-tailed *P* value = .19; Fig 2B). In the rPRESS algorithm, age > 49 years was proposed to favor the diagnosis of LMS over LM.^5^ In our series, the sensitivity, specificity, positive predictive value, and negative predictive value of this age cutoff were 62.5%, 55.56%, 55.56% (95% CI, 33.55% to 75.58%), and 62.5% (95% CI, 36.41% to 82.91%), respectively. This performance was poorer than the findings reported in the rPRESS study, where the sensitivity, specificity, positive predictive value, and negative predictive value were 93%, 65%, 45%, and 97%, respectively.^5^

### Low Mutation Burden in LM

Previous genomic studies of LM have identified *MED12* as the most frequently mutated gene in these lesions, but these studies did not report on other recurrent somatic mutations in these tumors.^12-14^ However, *MED12* mutations have been reported also in a subset of LMS tumors, and therefore these mutations cannot be used for a molecular distinction between LM and LMS.^15^ To investigate whether LM tumors might have recurrently mutated genes other than *MED12*, we performed whole-exome sequencing on 36 pairs of matched tumor and normal DNA specimens from patients with LM. We identified only one gene other than *MED12* that was affected by deleterious mutations in at least two patients with LM: the *ACLY* gene encoding ATP citrate lyase. Mutations in exon 2 of *MED12* were identified in 39% (14 of 36) of LM tumor specimens, and the *ACLY* gene was mutated in 6% (two of 36) of LM tumor specimens (Data Supplement). Mutations in these two genes were present in 15 of 36 patients, with a median of one mutation per tumor (range, 0-2 across all 36 tumors). All *MED12* mutations identified in our study were previously reported in the COSMIC database (COSMIC v84; accessed on November 12, 2018).^16^

Our results show that recurrent mutations other than in the *MED12* gene rarely occur in LM. Therefore, it is not practical to construct an LM-specific capture panel that could be applied for deep targeted sequencing using CAPP-Seq in patients with LM. Importantly, we did not detect in any LM case any of the mutations in tumor suppressor genes that are frequently mutated in LMS, such as *TP53*, *RB1*, *PTEN*, *ATRX*, *ATM*, and *ARID1A*.^6^ This indicates that mutations in these driver genes may be highly specific to malignant tumors such as LMS. Because LMs do not carry mutations in these tumor suppressor genes, we propose that detection of these mutations in plasma DNA of patients with uncertain diagnosis, using the CAPP-Seq LMS-specific panel developed in our previous study, may favor the diagnosis of LMS.

## DISCUSSION

The clinical utility of ctDNA profiling has been widely demonstrated in malignant tumors that harbor highly recurrent mutations.^7,8,17,18^ Currently, the two most frequently used sequencing-based approaches for ctDNA monitoring include deep targeted sequencing for ultrasensitive quantitative analysis of point mutations, such as CAPP-Seq, and shallow whole-genome sequencing for the detection of CNAs. We have previously combined both methods to monitor ctDNA in patients with LMS,^6^ which allowed for a comprehensive monitoring of a broad spectrum of tumor-specific markers in plasma DNA. In the current study, we show that LM-derived CNAs can be detected in plasma DNA in a substantial portion of patients. However, it is not practical to design an LM-specific capture panel for detection of point mutations by deep targeted sequencing of ctDNA, because we identified only two recurrently mutated genes in a low percentage of 36 LM tumors.

Although ctDNA released from malignant tumors has been well characterized in multiple types of cancer, ctDNA derived from benign lesions has not been extensively studied. An incidental finding of LM-derived DNA in the circulatory system has been reported in pregnant women undergoing noninvasive prenatal testing (NIPT). Dharajiya et al^19^ reported unexpectedly abnormal NIPT profiles in 55 of 450,000 pregnant women tested for fetal aneuploidy; 20 of these 55 women were known to have had LM at the time of NIPT. But the genomic profile of a matching tumor was examined only in a single patient from this group to confirm that the abnormal levels of cell-free DNA were indeed derived from this LM.^19^ Although these incidental findings indicated that LM can shed DNA into the circulatory system, the overall sensitivity of shallow whole-genome sequencing for the detection of LM-derived ctDNA has not been investigated. In the current study, by prospective analysis of plasma and tumor samples of 12 patients with LM, we found ctDNA in 50% of these patients, which is a comparable detection rate to the current sensitivity of ctDNA detection in many stage I cancers.^20,21^ Importantly, the NIPT assays used for detecting fetal abnormalities usually focus solely on screening for monosomy or trisomy of chromosomes 13, 18, and/or 21. In the current study, instead of focusing only on selected chromosomes, we performed a genome-wide analysis in which we aimed to detect not only chromosome-wide aberrations but also small focal aberrations. We performed shallow whole-genome sequencing at approximately 0.1× genome-wide coverage; however, we expect that deeper sequencing would allow for the detection of ctDNA in a higher percentage of patients with LM. On the basis of previously published studies, we assume that an approximately two-fold increase of coverage may result in improving the reliability of the calls, by reducing the number of false-positive calls and increasing the number of true-positive calls.^22^ Moreover, most of the patients included in our study had multifocal disease, and we expect that profiling all nodules from these patients would reveal a wider spectrum of CNAs and thus allow for the detection of overlapping aberrations between plasma and tumor DNA in a higher percentage of patients with LM.

It must be noted that the development of a reliable assay for the distinction between LM and LMS is challenged by the great difference in prevalence of these two entities. Given the very low prevalence of LMS and the very high prevalence of LM (prior probability of LMS *v* LM in the range of 0.12% to 1.9%),^23^ the highest positive predictive values and negative predictive values could be achieved for tests that would confirm the diagnosis of LM or exclude the diagnosis of LMS, respectively. For example, assuming that the prevalence of LMS is 0.12% and that the test would have a high sensitivity and specificity of 95%, the positive predictive value for any positive result indicating LMS would be only 2%, while the negative predictive value of a negative result would be 99.99%. On the basis of the genomic profiles of LM and LMS, we propose that it may be more practical to apply an assay that would rule out the diagnosis of LM. This could be possible by applying CAPP-Seq for detection of mutations in tumor suppressor genes that are frequently mutated in LMS but have never been reported in LM. Regardless, given the statistical considerations on the prevalence of LM and LMS, validation of such assay in the clinical setting may be challenging and would require a very large prospective study.

In summary, we demonstrate in the current study that a substantial portion of LMs do shed DNA into the circulatory system. These findings provide an opportunity to develop a noninvasive test for distinction between LM and LMS on the basis of ctDNA in plasma. However, the low complexity of genomic profiles of LM and the profound differences in the prevalence of LM and LMS pose significant challenges in development of such assays. We propose that a clinical benefit may be derived from ctDNA-based detection of point mutations and CNAs in selected tumor suppressor genes that are frequently affected in LMS and to date have not been reported in LM.
